# The role of dosimetry and biological effects in metastatic castration–resistant prostate cancer (mCRPC) patients treated with ^223^Ra: first in human study

**DOI:** 10.1186/s13046-021-02056-9

**Published:** 2021-09-06

**Authors:** Rosa Sciuto, Sandra Rea, Sara Ungania, Antonella Testa, Valentina Dini, Maria Antonella Tabocchini, Clarice Patrono, Antonella Soriani, Valentina Palma, Raffaella Marconi, Lidia Strigari

**Affiliations:** 1 Nuclear Medicine Unit, IRCCS Regina Elena National Cancer Institute, Rome, Italy; 2 Laboratory of Medical Physics, IRCCS Regina Elena National Cancer Institute, Rome, Italy; 3 Division of Health Protection Technologies, ENEA Casaccia Research Center, Rome, Italy; 4 National Center for Innovative Technologies in Public Health, Istituto Superiore di Sanità, Rome, Italy; 5 INFN, Rome, Italy; 6 Present address: Department of Medical Physics, IRCCS Azienda Ospedaliero-Universitaria di Bologna, Bologna, Italy

**Keywords:** Dosimetry, Biological effects, ^223^Ra

## Abstract

**Background:**

^223^Ra is currently used for treatment of metastatic castration resistant prostate cancer patients (mCRPC) bone metastases with fixed standard activity. Individualized treatments, based on adsorbed dose (AD) in target and non-target tissue, are absolutely needed to optimize efficacy while reducing toxicity of α-emitter targeted therapy. This is a pilot first in human clinical trial aimed to correlate dosimetry, clinical response and biological side effects to personalize ^223^Ra treatment.

**Methods:**

Out of 20 mCRPC patients who underwent standard ^223^Ra treatment and dosimetry, in a subset of 5 patients the AD to target and non-target tissues was correlated with clinical effects and radiation-induced chromosome damages. Before each ^223^Ra administrations, haematological parameters, PSA and ALP values were evaluated. Additional blood samples were obtained baseline (T0), at 7 days (T7), 30 days (T30) and 180 days (T180) to evaluate chromosome damage. After administration WB planar ^223^Ra images were obtained at 2–4 and 18–24 h. Treatment response and toxicity were monitored with clinical evaluation, bone scan, 18F-choline-PET/CT, PSA value and ALP while haematological parameters were evaluated weekly after ^223^Ra injection and 2 months after last cycle.

**Results:**

1. a correlation between AD to target and clinical response was evidenced with threshold of 20 Gy as a cut-off to obtain tumor control; 2. the AD to red marrow was lower than 2 Gy in all the patients with no apparently correlation between dosimetry and clinical toxicity. 3. a high dose dependent increase of the number of dicentrics and micronuclei during the course of ^223^Ra therapy was observed and a linear correlation has been found between blood AD (BAD) and number of dicentrics.

**Conclusions:**

This study provides some interesting preliminary evidence to be further investigated: dosimetry may be useful to identify a more appropriate ^223^Ra administered activity predicting AD to target tissue; a dose dependent complex chromosome damage occurs during ^223^Ra administration and this injury is more evident in heavily pre-treated patients; dosimetry could be used for radioprotection purpose.

**Trial registration:**

The pilot study has been approved from the Ethics Committee of Regina Elena National Cancer Institute (N:RS1083/18–2111).

**Supplementary Information:**

The online version contains supplementary material available at 10.1186/s13046-021-02056-9.

## Background

Radium-223 (^223^Ra) dichloride (Alpharadin®) is the first targeted α-therapy approved by FDA for the treatment of patients with metastatic castration resistant prostate cancer patients (mCRPC) with symptomatic bone metastases and no known visceral metastatic disease. ^223^Ra targets bone metastases with high linear energy transfer (LET), short range (< 100 μm) α-particles.

Several clinical studies published in recent years suggest that ^223^Ra may provide a new standard of care for patients with mCRPC and bone metastases improving overall survival and reducing the time to the first symptomatic skeletal event with a very low toxicity [1–3].

The approved regimen used worldwide consists of a course of six ^223^Ra injections with a standard activity of 55 kBq/kg every 4-week [4–6].

However, mCRPC condition represents a very broad spectrum of disease and a standard activity may not be appropriate in all cases. Radiopharmaceutical treatment cannot be considered as a pharmacological treatment and schedules based on body weight are not suitable for getting the best therapeutic ratio [7]. Fixed schedules may result in over or under dosage limiting efficacy or increasing toxicity, mainly in α-emitter targeted therapy that is potentially highly effective but also quite toxic [8].

Efficacy and toxicity are due to adsorbed dose (AD) that is related to individual ^223^Ra biodistribution in target, i.e. bone metastases, and non-target tissues.

The pharmacokinetic and dosimetry of ^223^Ra in selected patients have been reported with promising results [9, 10]. Wide difference in radium uptake and biodistribution has been evidenced in clinical use while the retention time of the ^223^Ra in the body has been demonstrated to range from 11 to 70% after 30 days from the first administration.

To date however, there are no published studies aiming at modifying the administered activity according the patients and tumor features using dosimetry. Moreover, several studies indicated that in experiments with alpha particles, more cells were damaged than were traversed by alpha particles [11, 12]. Radiobiological mechanisms of α-emitters could therefore play a relevant role for haematological toxicity or secondary radiation induced tumors and should be deeper investigated.

The understanding of the physical and biologic factors that impacts response and toxicity in non-target tissues is essential to avoid the risk that α-emitters may be abandoned before they have been properly tested in the clinic.

This is a first translational prospective pilot study in humans aimed at improving α-emitters radiobiological model knowledge and demonstrating the potential applicability of dosimetry to evaluate health risks associated with α-particle exposure. For this purpose, the AD to target and non-target tissues of standard 223Ra treatment in a group of 5 mCRPC patients was correlated with clinical effects and with the radiation-induced chromosome damage in peripheral blood lymphocytes (PBLs) representing a non-target tissue.

## Methods

### Study design

The study design is an observational, prospective, first in human clinical trial evaluating the relationships between dosimetry and efficacy and safety of ^223^Ra standard treatment on a cohort of 20 mCRPC patients. Preliminary data observed in a subset of 5 patients in which biological effects were also tested are reported in this paper. The clinical trial was conducted in accordance with the Declaration of Helsinki and good clinical practices guidelines and each patient provided a specific written informed consent. The protocol was approved from the Ethics Committee of Regina Elena National Cancer Institute, Rome, Italy (number: RS1083/18–2111).

The primary endpoint of this pilot study was to evaluate the predictive value of dosimetry on target tissues. Further endpoints included the evaluation of the following parameters: the patient based and lesion-based response; safety and haematological toxicity; the chromosome damage, in terms of Dicentric (DC) and micronuclei (MN) induced in PBLs during the course of therapy for assessing non-target tissue effects; the predictive value of dosimetry on non-target tissues with respect to haematological toxicity; the correlation between chromosome damage in PBLs and haematological clinical toxicity.

The timeline of the study includes different steps and activities: 1. patients’ enrollment based on pre-treatment images; 2. treatment with six ^223^Ra injections and related blood samples collections and images acquisitions; 3. dosimetry; 4. chromosome damage evaluation in PBLs; 5. post treatment images and follow-up. These activities are summarized in graphical abstract and detailed in the following paragraphs.

### Patients

All patients were previously evaluated for the entry to the study in a multidisciplinary setting. Baseline examination included history, clinical examination as well as baseline blood tests to evaluate PSA, alkaline phosphatase level test (ALP), haematological parameters, white blood cells (WBCs), red blood cells (RBCs), Hemoglobin (Hgb) and platelets (PLTs) values and imaging: ^18^F-choline PET/CT (FchPET), ^99m^technetium methylene diphosphonate *(*^*99m*^*Tc-MDP*) bone scan (BS) and CT of chest, abdomen and pelvis). Eligibility required at least two documented symptomatic bone metastases under androgen ablation therapy; Eastern Cooperative Oncology Group score 0–2, life expectancy > 6 months, age > 18 years, adequate hematologic function and the availability of nuclear imaging, i.e. FchPET and BS performed in our Institute less than 1 month before the enrolment.

### Treatment

Each enrolled patient received monthly i.v. ^223^Ra injections with a standard activity of 55 kBq/kg for a maximum of six cycles. Before each ^223^Ra administration a baseline blood samples were collected.

Additional series of blood samples were also obtained before the first ^223^Ra administration (T0) and afterwards at 7 days (T7), 30 days (T30) and 180 days (T180) to evaluate chromosome damage in PBLs.

### Image acquisition and dosimetry

The ^223^Ra activity was measured using the radionuclide calibrator PET DOSE (Comecer) following the procedures in [13, 14]. For each patient, activity time curves were determined using antero-posterior and postero-anterior 30 min planar images acquired at 2–4, 18–24 h and 7 days after the each ^223^Ra administration. Technical details about acquisition and correction factors to be applied to the images were reported in additional supplementary data (S1: Fig. S1 and S2: supplementary notes).

**Fig. 1:**
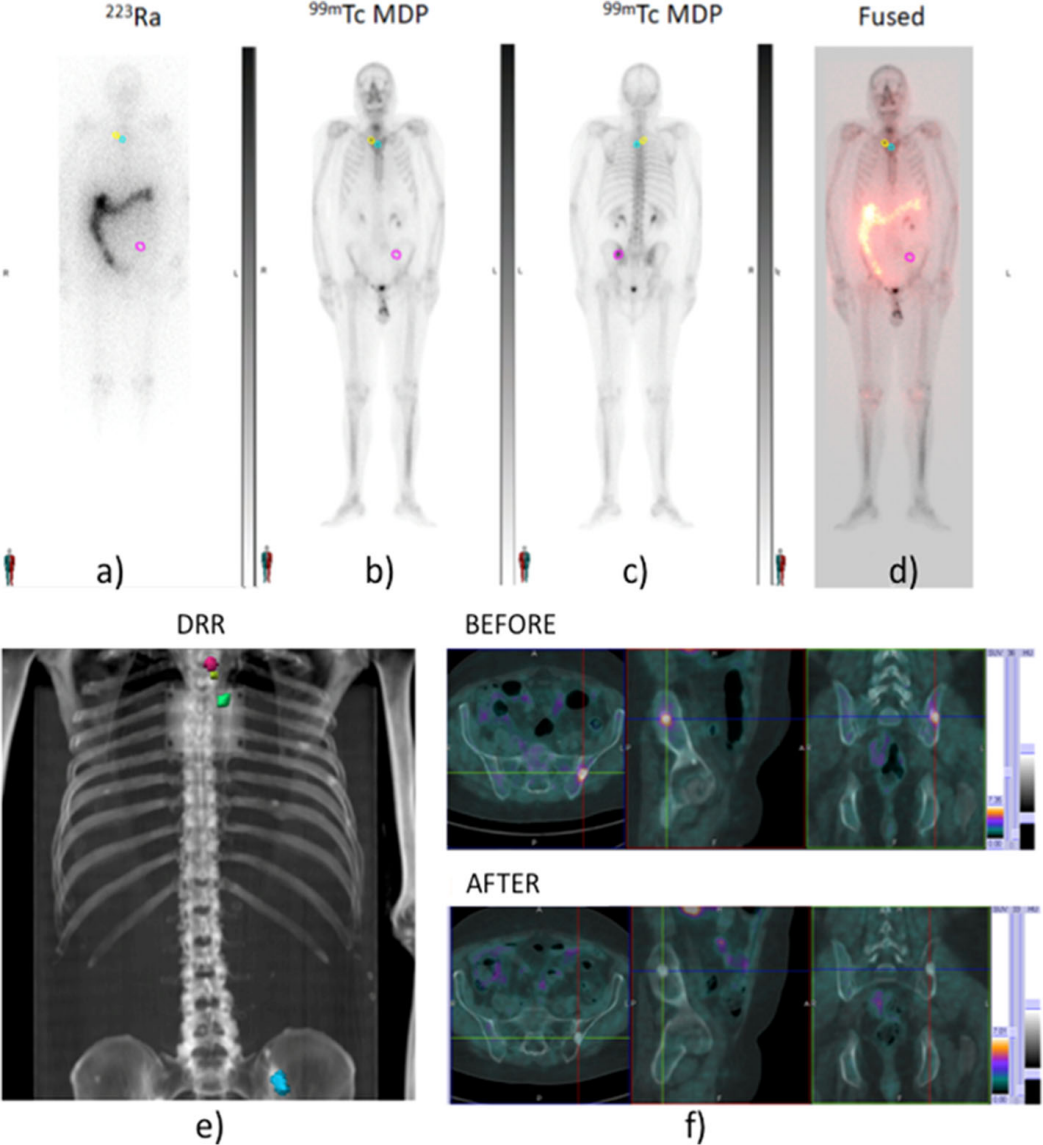
a) anterior planar ^223^Ra image**,** b) anterior, c) posterior planar ^99m^Tc-MDP images; d) ^223^Ra and ^99m^Tc-MDP co-registered images; e) ROIs identified on the calculated DRR; f) target volumes into FchPET images before and after treatment with ^223^Ra

Planar ^223^Ra images (Fig. 1**a**) were co-registered to the basal ^99m^Tc-MDP bone scan study (Fig. 1**b,c**) and with the Whole-Body scans using MIM 6.1.7 (MIM Software Inc., Ohio) (Fig. 1**d**). Regions of interest (ROIs) were delineated and transferred onto the ^223^Ra static images and onto the calculated transmission images (Digital Reconstructed Radiograph, i.e.DRR) (Fig. 1**e**). Both ^99m^Tc-MDP and ^223^Ra planar images were also visually compared with activity distribution in co-registered FchPET images before and after treatment (Fig. 1**f**) to identify the functional target volume. Exposure rate was measured at 5 cm, 1 and 2 m from the patient surface as described in D’Alessio et al. [15] at 1, 2–4, 24 and 48 h, and at 7 days post-injection. The time scheduling was modified according to patient compliance.

The blood samples for dosimetry were collected at 2–4 and 18–24 h after injection and measured using a well counter of Atomlab™ Gamma Counter (Biodex Medical Systems, Inc).

Tumor and red marrow (RM) AD was calculated using IDAC Dose 2.1 [16]. Time integrated activity curve (TIAC) was included for RM into IDAC Dose 2.1.

An RBE value of 5.5 were used to convert the absorbed to the equivalent dose for tumor and organs at risk (OARs), in agreement to [17, 18]; while a relative biological effectiveness (*RBE*) of 20 was assumed for AD to blood according to [11].

### Chromosome damage evaluation

The blood samples for evaluation of chromosome damage in PBLs were collected: 1) before the first administration (T0), 2) at day 7 after administration (T7), 3) at day 30, immediately before second administration (T30), 4) at day 180, after the end of treatment (T180). Chromosome damage, in terms of DC and MN induction, was evaluated for each time points accordingly to standard protocols [19].

Evaluation of DC and MN in exposed individuals’ PBLs is the most commonly used biological dosimetry approach.

DC originates from an asymmetric exchange between the centromeric pieces of two broken chromosomes which in its complete form is accompanied by a fragment composed by the acentric pieces of these chromosomes. This method, after more than 50 years from its set up, is still considered as the “gold standard” of the biological dosimetry, the dicentric induction being considered radiation-specific.

The in vitro cytokinesis-block micronucleus assay has been used for biological dosimetry since 1985 [20]. MN are small nuclei that form whenever a whole chromatid/chromosome or chromatid/chromosome fragments are not incorporated into one of the daughter nuclei during cell division. MN are not as radiation specific as dicentrics, since they may be induced either by clastogenic chemicals or aneugenic agents.

As for the dicentric assay, 200 metaphases were scored for each experimental point: both DC and centric rings were included in the scoring of chromosomal aberrations. An average of 3000 binucleated cells were analyzed for the MN induction.

These results on chromosome damage were analyzed versus the AD to blood to establish a possible correlation between the ^223^Ra therapy and the genetic damage induced in a non-target tissue.

### Treatment response and toxicity

Treatment response was monitored with clinical evaluation, BS, FchPET, PSA value and ALP both interim (before the fourth cycle) and at end-treatment within 3 months after the last cycle. Haematological parameters were monitored every week after ^223^Ra injection and 2 months after last cycle of ^223^Ra treatment to evaluate toxicity. Clinical follow-up every 3 months was also extended until to progression or death. Efficacy was evaluated both on per-patient analysis overall clinical response (OCR) and on per- lesion analysis target tissue response (TTR) within 3 months after the end of treatment. OCR was evaluated in a multidisciplinary setting and graded as responder if a combination of measured parameters improved (PSA, ALP, imaging and clinical condition).

Lesion based response at 3 months was also evaluated to define TTR on imaging distinguishing between complete (CR), partial (PR), stable

(SD) and progressive disease (PD) according to PERCIST criteria [21]. The occurrence of post-treatment grade 3/4 haematological toxicities up to 6 months after last administration of ^223^Ra was considered as adverse events or Serious Adverse Events (SAEs) according to National Cancer Institute-Common Terminology Criteria for Adverse Events (NCI-CTCAE), version 4.03. Non-haematological toxicity was also evaluated and skeleton-related event, fatigue, general health deterioration, spinal cord compression were reported.

### Statistical analysis

Categorical variables were presented as number with percentage in descriptive tables, while continuous variables were presented as median (range) or mean and standard deviation as appropriate. Parameters without a normal distribution were logarithmically converted.

Two independent groups were tested by the Student’s t-test or Mann-Whitney test as appropriated. Correlations between variables were investigated by the Pearson correlation coefficient. Prognostic dosimetric or and clinical/pathological variables on tumor control as well as haematological toxicity were analyzed. The performance of number of dicentrics versus AD to blood was evaluated using Bland-Altman analysis. *P* value < 0.05 was considered statistically significant.

## Results

### Treatment response and toxicity

The baseline clinical characteristics of the 5 mCRPC patients are reported in Table 1. The median age was 72 years (range 60-84 yrs.). The median basal PSA was 42.30 ng/mL (range 6.74–757 ng/mL) while median ALP value was 89 U/L (range 51–418 U/L).

**Table. 1:** Patients’ characteristics at study entry

Pt #	Age	Weight	GleasonScore	PSA	ALP	ECOG status	N. of BoneMets	Haemoglobin level	Testosteronlevel	BM delay	PreviousTreatment
	(Yrs)	(Kg)		(ng/ml)	(U/L)			(g /dl)	(ng/ml)	(months)	
**1**	84	97	9	3.82	51	0	2	13.60	0.03	116	RT
**2**	72	72	7	42.30	59	2	> 20	13.20	0.03	48	ABI-ENZA
**3**	78	78	7	1161	170	2	> 20	12.80	0.03	48	ABICT (1 line)
**4**	60	60	9	757.00	418	2	10–20	10.40	0.03	18	ABI-ENZARTCT (2 lines)
**5**	60	60	7	6.74	89	0	4	15.80	0.09	0	ABI - ENZA

Legend: *ECOG* Eastern Cooperative Oncology Group, *BM delay* time from initial prostate cancer diagnosis to bone metastases diagnosis, *ABI* abiraterone, *ENZA* enzatulamide, *CT* chemotherapy, *RT* bone targeted radiotherapy, *PSA* prostate-specific antigen, *ALP* alkaline phosphatase

Patients who had been treated with chemotherapy prior to ^223^Ra appeared to have poorer baseline characteristics than those who had not (Table 1), presenting a higher ECOG performance status, a higher bone involvement with > 20 metastatic lesions, higher median levels of PSA (132.0 vs 40.2 ng/mL) and ALP (162.0 vs 115.0 U/L).

All patients received at least 3 cycles of ^223^Ra and three patients (60%) received all 6 planned cycles (pt. 1, 2 and 5). Only two out of 5 patients were considered clinical responders (Table 2).

**Table. 2:** clinical response and toxicity

Pt.	# of ^**223**^Ra administrations	Patient response (Imaging + marker + clinical status)	Toxicity grade	Toxicity type	PFS (months)	PFS event	OS (months)	Cause of death
1	6	Responder	0	–	13	No Disease progression	13	No tumor related
2	6	No Responder	0	–	18	Disease progression	19	Disease progression
3	3	No Responder	3	Anemya	6	Disease progression	6	Disease progression
4	3	No Responder	3	Anemya	3	Disease progression	6	Disease progression
5	6	Responder	0	–	24	No Disease progression	24	Alive

Median survival from last treatment was 13 months while median progression time from last treatment was 6 months ranging from 3 up to 18 months. At progression time two patients received only palliative cure (pt. 3 and 4), one patient re-challenged to chemotherapy (pt. 2), one patient received external beam radiotherapy (EBRT, i.e. pt. 5) and one patient needed no further treatment until death (pt. 1).

**Fig. 2:**
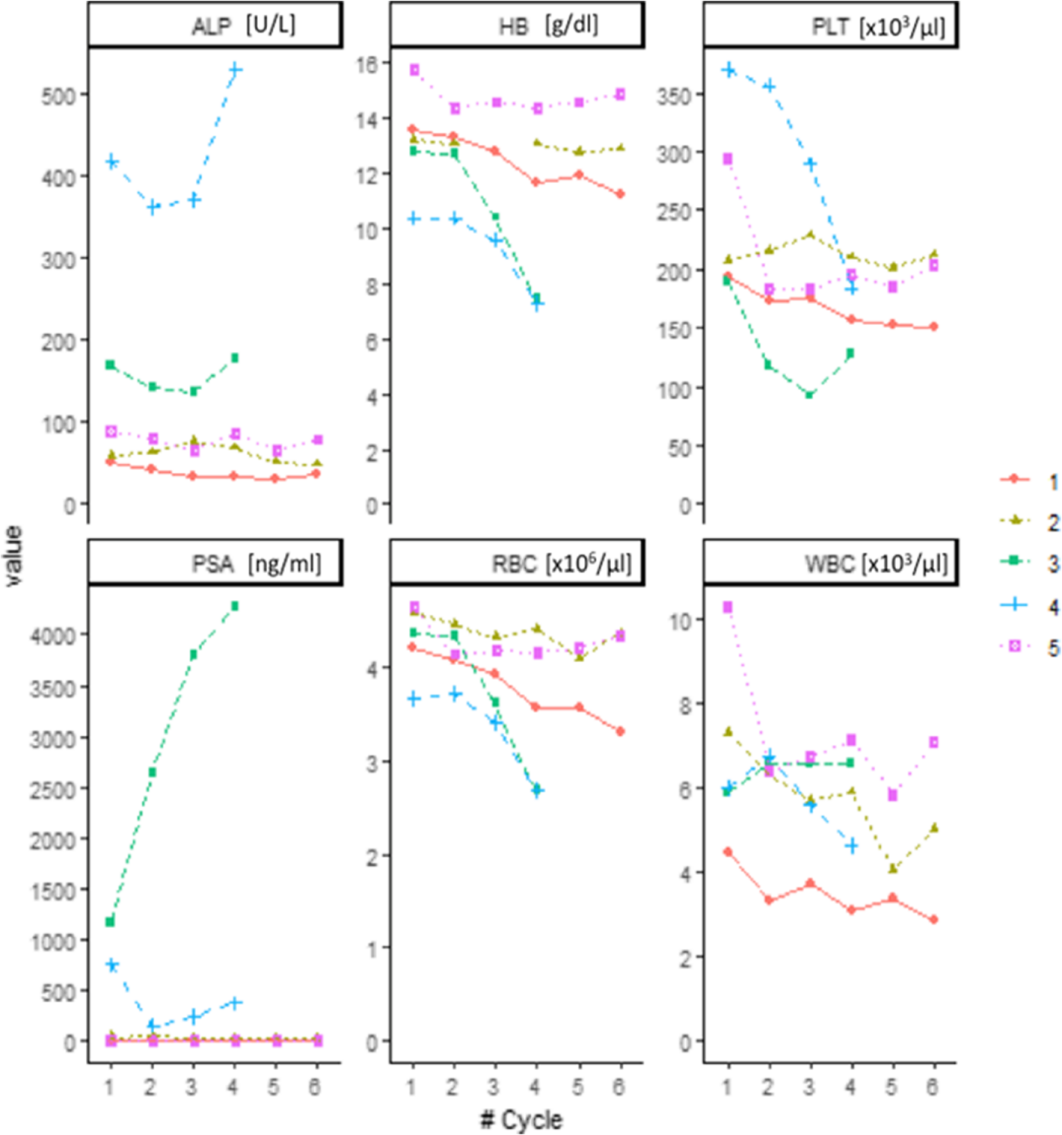
Behavior of markers of disease burden (ALP and PSA) and haematopoietic impairment (HB, PLT, RBC, WBC) before of each ^223^Ra administration. Values are reported as symbols, while lines are used only to connect all the patient values. Numbers indicate the enrolled patients

A decrease in ALP was observed in three patients while PSA decreased in all patients out one (pt. 3), see Fig. 2.

The enrolled patients showed a wide difference in clinical presentation in terms of markers of disease burden (PSA and ALP) and hematopoietic impairment (WBC, RBC, HB, PLT). On per-patient analysis, the overall clinical response was observed only in two patients (pt. 1 and 5) in which both performance score, PET imaging at 3 months and PSA/ALP values improved. The other three patients were considered non-responders (Table 2).

Severe anemia requiring blood transfusions after three cycles of treatment was observed in the two patients who had received prior chemotherapy (patients 3 and 4). Mild and transient thrombocytopenia was observed also in one patient (pt. 3) while mild leukopenia with lymphopenia was observed in patient 1. Haematological values during treatments are shown in the Fig. 2**.** No patients experienced any symptomatic skeletal event during or after treatment and all out one patient reported a decrease in pain from pre-treatment baseline.

### Objective response and target dosimetry

To define lesion-based response, 20 target lesions were identified in the 5 patients. The 3-month lesion-based response at PET imaging was PD (61%), SD (28%), PR (28%), CR (17%). The absence of statistically significant correlation between AD to the lesions and either the administered activity or the activity administered per kg was observed.

**Fig. 3:**
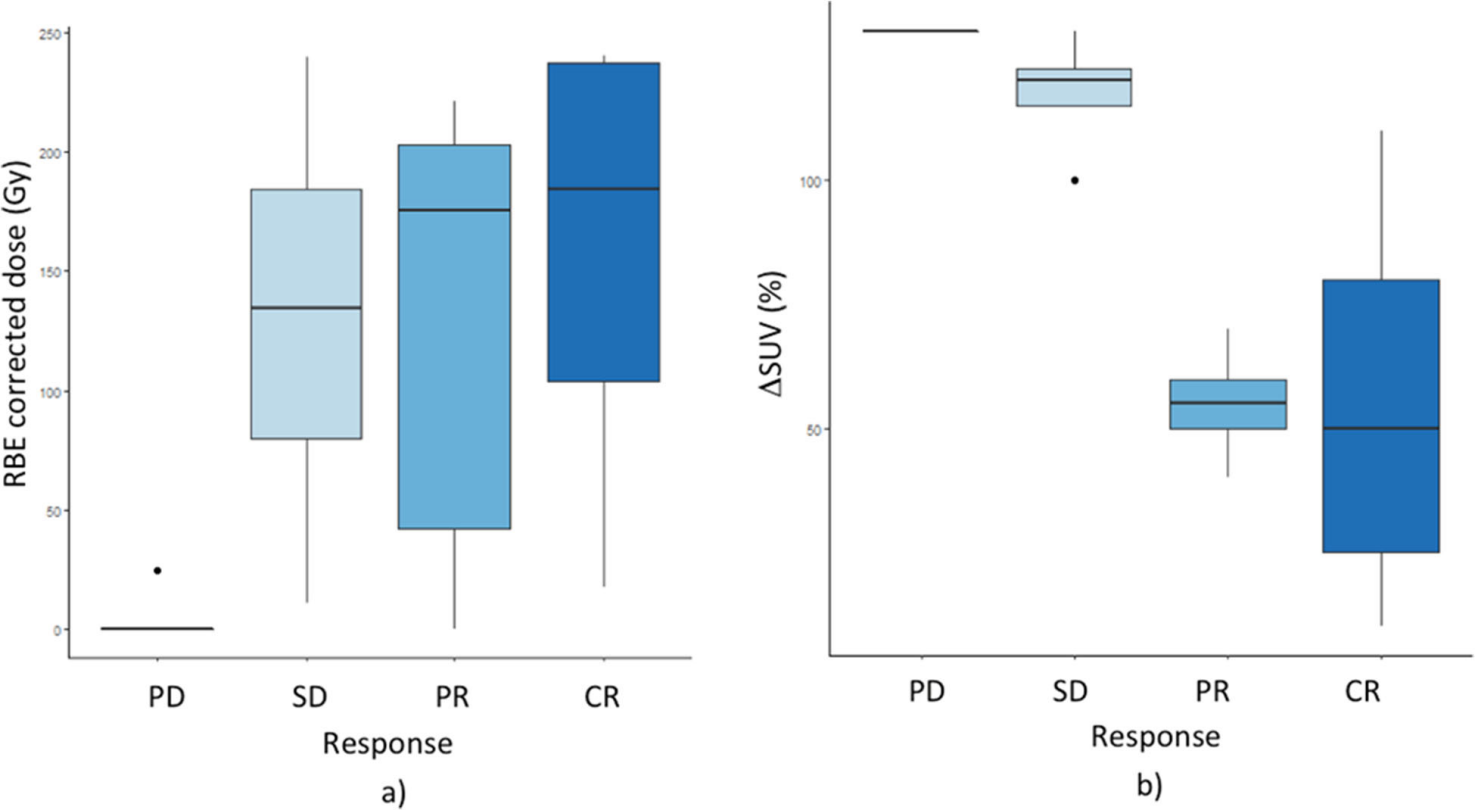
**a)** RBE corrected absorbed dose and **b**) SUV variation versus target objective response

Based on PET imaging, target volumes ranged from 0.58 to 40 ml. Target doses ranged from 0.001 Gy to 43.7 Gy (median 30.1 Gy). An AD response relationship for target lesions has been observed with a threshold of 20 Gy. The RBE corrected AD versus the target objective response is shown in Fig. 3**a**. The SUV (standard uptake value) variation according to the lesion response is also reported in Fig. 3**b**.

### Toxicity and non-target dosimetry

**Fig. 4:**
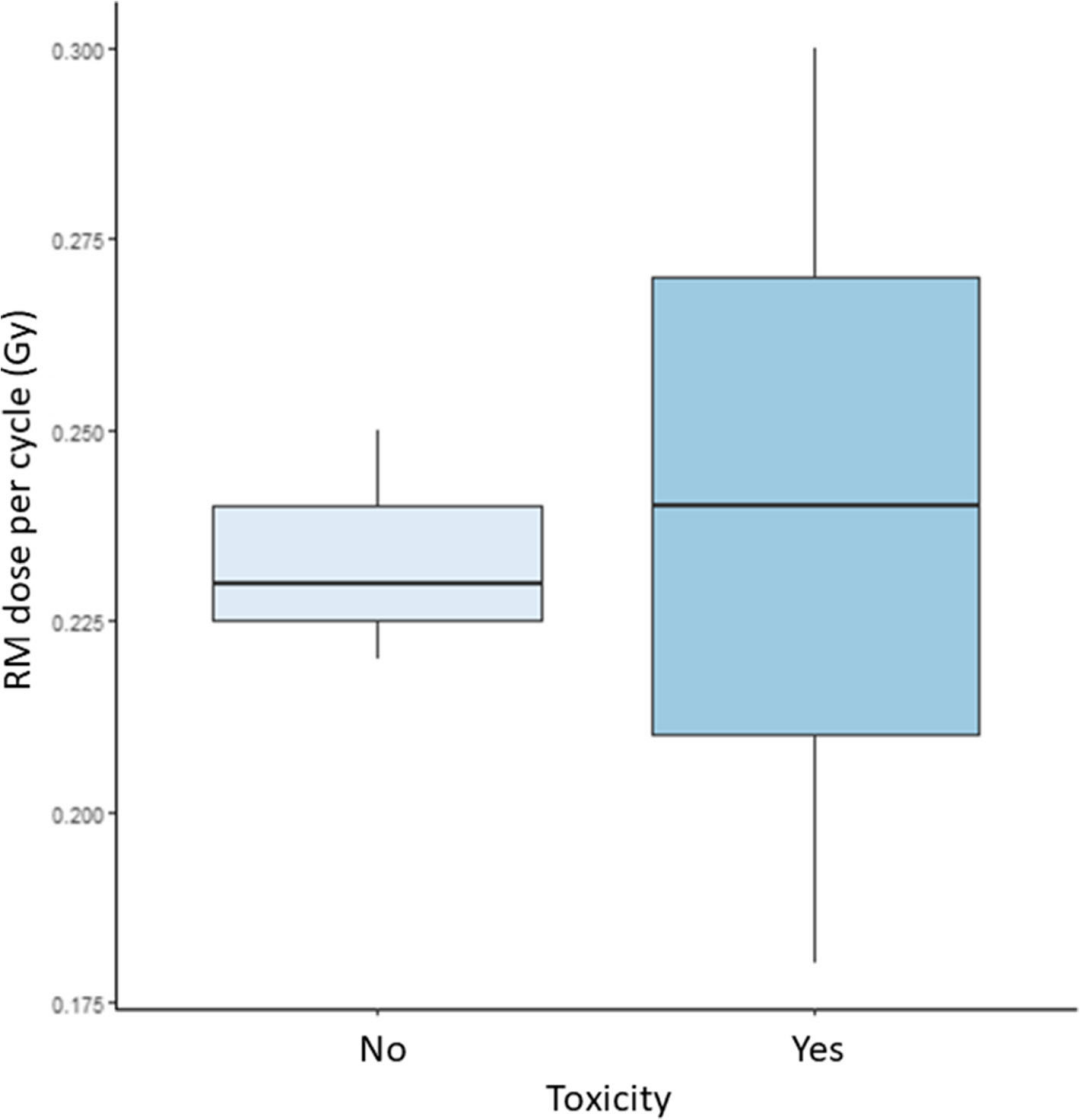
RM absorbed dose at the first cycle in patients with (Yes) and without (No) any haematological toxicity

The AD to RM was < 2 Gy in all patients. Figure 4 shows the RM AD at the first cycle in patients with (2 patients) and without (3 patients) any haematological toxicity (i.e. anemia). The absence of statistically significant correlation between RM absorbed dose and haematological toxicity was observed. In addition, the haematological toxicity was observed in 2/5 patients although the activity administered per kg was the same in the whole groups of patients. The two patients manifesting grade 3 haematological toxicity were previously treated with CHT**.**

### Chromosome damage

The average dicentric frequency found in T0 is very high (0.073 ± 0.008) compared to the general background of control subjects (0.001/0.002). This is likely due to previous treatments (particularly, 2 patients received external beam radiotherapy before ^223^Ra treatment).

**Fig. 5:**
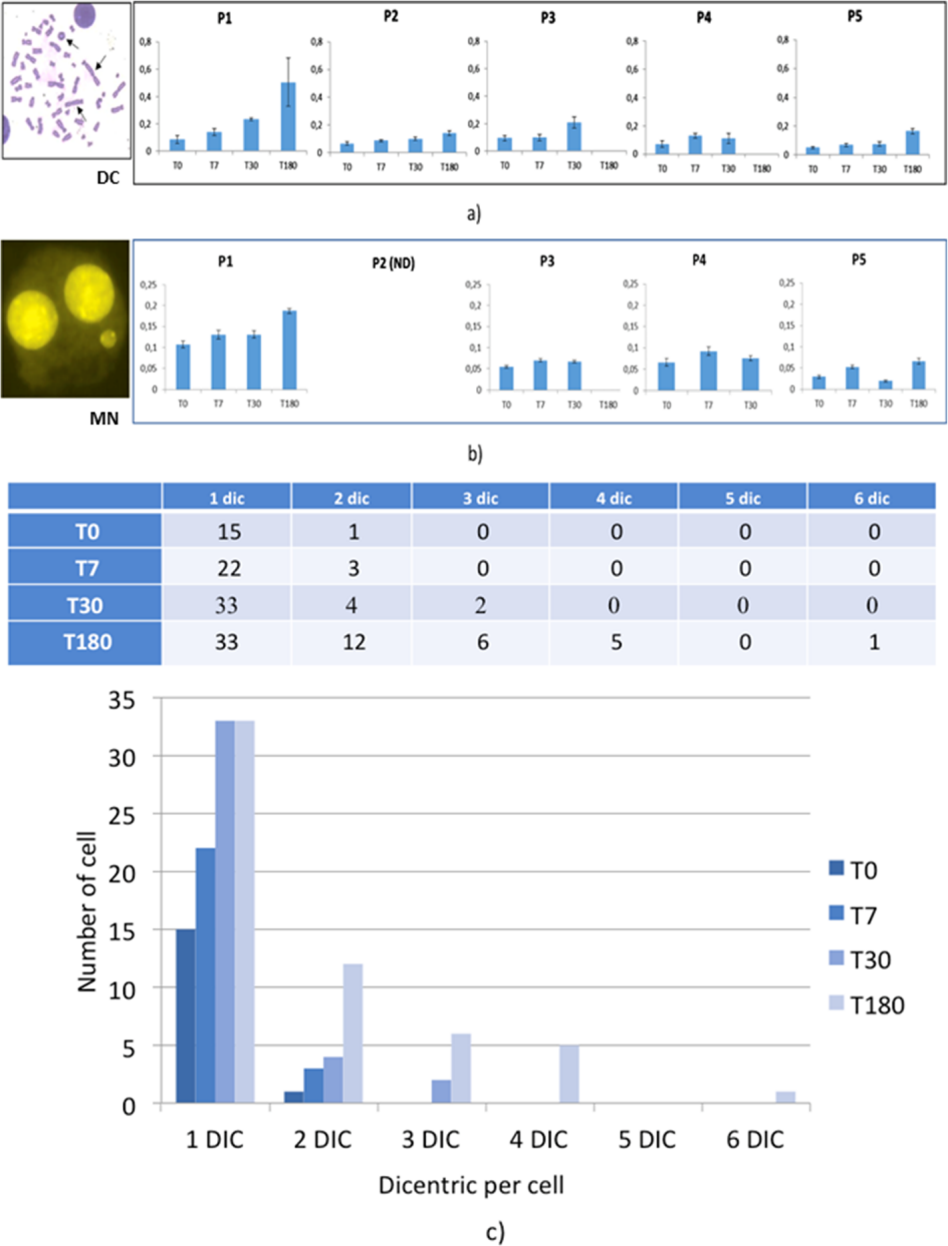
a) Frequencies of DC; b) MN at different time points for the investigated patients and c) dicentric distribution in PBLs of patient 1: the number of cells with one or more dicentrics (out of 200 metaphases) is represented for each experimental point; a similar trend was observed in the other patients

In all patients, the average DC frequencies show a dose-dependent increase during the course of ^223^Ra treatment (T7 = 0.105 ± 0.013; T30 = 0.146 ± 0.032) reaching the highest level at the completion of the therapy (T180 = 0.27 ± 0.118) (Fig. 5**a**). In patients 1 and 3, a sharp increase of DC was observed between T7 and T30, even though no additional ^223^Ra administration occurred in this interval**.**

Moreover, a progressive increase of the complex chromosome damage (number of cells with 2 or more DCs) is registered over the course of the ^223^Ra therapy in all patients (Fig. 5**c**)**.**

Overall, the results show an increase in the average frequency of MN as treatment time increases up to a maximum of 0.126 ± 0.061 at T180 with a slight decrease at T30 (0.0731 ± 0.023) corresponding to the interval between the two treatments (Fig. 5**b**).

**Fig. 6:**
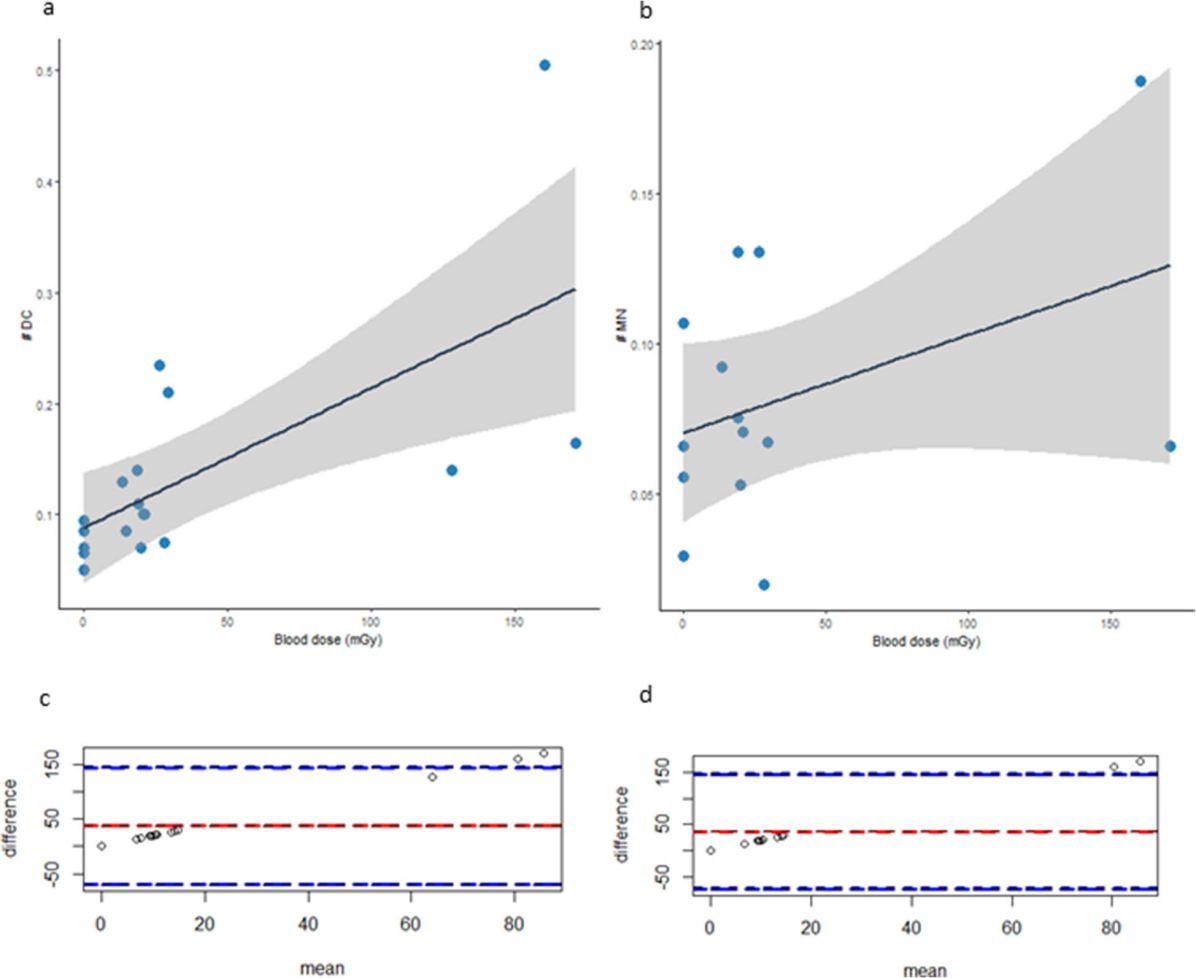
Number of DC (a) and MN (b) observed in the PBLs (non-target-tissue) plotted against the AD to blood. Solid line represents the fitted curve and the grey area the 95% confidence interval. Bland-Altman plots of difference in number of c) DC and d) MN and the AD to blood. Red line represents the average between the two methods and blue dotted line indicate the 95% limits of agreement for comparison

The DC and MN frequencies observed in PBLs (non-target-tissue) have been plotted against the AD to blood (Fig. 6a-b). A linear correlation has been found between AD to blood and number of DC (Pearson’s product-moment correlation cor = 0.658, *p*-value = 0.003) but not with the number of MN (cor = 0.41, *p-*value = 0.14).

Bland-Altman plots of difference in DC and MN frequencies versus the AD to blood (Fig. 6**c-d**) showed good correlation and agreement between the two methods, with few samples falling outside the 95% limits of agreement for each comparison (average difference ± 1.96 standard deviation of the difference).

The samples falling outside the 95% limits correspond to calculated doses after therapy, so the disagreement could be due to the fact we calculated the dose at the end of therapy using the images obtained at the first cycle. This can be considered an issue of our approach. In addition, the absence of statistically significant correlation between RM absorbed dose and haematological toxicity was registered.

## Discussion

Targeted α-emitter therapy represents the future of nuclear medicine therapy and many novel radiotracers are under evaluation [22]. To improve knowledge of AD and biological effects of these treatments both on target and healthy tissue is therefore mandatory.

The aim of this study is to correlate dosimetry, clinical response and biological side effects to optimize and personalize the ^223^Ra treatment schedule.

Firstly, our results evidenced a correlation between target dose and clinical response. A threshold of 20Gy was identified as a cut-off to obtain tumor control. This supports the possibility of improving treatment efficacy through dosimetric estimation of the activity to administer and personalization of ^223^Ra schedules fulfilling the requirement of Directive 2013/59.

Moreover, the observed correlation between SUV variation in FChPET and response demonstrated that ^223^Ra needs multimodal imaging to identify the biological target volume.

The second point addressed was the analysis of clinical toxicity and related AD and biological factors. Our results confirm that anaemia is the most represented AE related to ^223^Ra treatment and the main reason for treatment interruption [23]. AD apparently does not seem to correlate with clinical toxicity. Indeed, despite the AD to RM resulted < 2 Gy in all patients two of them presented severe anemia.

Also, it is worth noting that patient #3 and #4 did not complete treatment due to severe side effects, although the estimated dose after first cycle is similar to one received from other patients. This suggests that a potential lower dose threshold level for haematological toxicity should be adopted for heavily chemo treated patients to prevent toxicity from α-emitters. Further studies are needed to highlight this issue. Looking at the Fig. 5 of Andersson paper [24] - based on Taprogge, J. et al. paper [25]- the RM absorbed dose is ~ 13 and ~ 11 mGy/MBq, with or without considering the progenies biokinetic model, respectively. This means that our calculation might overestimate the RM dose of about 18% without considering the progenies biokinetic model. Moreover, the RM absorbed dose for intravenous Ra-223 based on the ICRP Publication 137 [26] for male worker is ~ 30 mGy/MBq including all progenies of Ra-223, while it was 34 and 92 based on Lassman and Nosske [27] or Yoshida et al. [28], respectively.

The equivalent dose to OARs per injected activity was calculated considering an RBE factor of 5 included in the range of RBE calculated using in vitro and in vivo studies for other α-particle therapy [29]. Stephan [11] using ^224^Ra suggested a radiation weighting factor of 20 for α-radiation for radioprotection issues. The appropriate RBEs to be used for response/toxicity/secondary effects estimation must be still determined and this requires more clinical data.

The prevention of toxicity is of paramount importance for this treatment representing the unique for which an improvement of OS has been reported. Regardless the adopted RBE value, lower RM AD constraints for patients undergoing chemotherapy should be highlighted.

Finally, this is the first in human study evaluating biological effects and chromosome damage after ^223^Ra administration. Unfortunately, the chromosome damage induced by internal radiation exposure is a difficult field of investigation.

Internal exposures are generally more complex to manage than external exposures. As highlighted by a recent review [30], the local absorbed dose rates (generally higher in the tumor and lower in the OARs) follow complex patterns which depend on the physical, chemical and metabolic properties of the radionuclide(s) and on patients’ anatomical characteristics. The irradiation of the body is spatially inhomogeneous, potentially prolonged over large periods and variable over time; thus, internal exposures become particularly problematic for biological dosimetry methods.

Therefore, even if the induction of chromosome aberrations is generally observed in PBLs of subjects internally contaminated, many factors must be considered to derive a meaningful estimate of radiation dose to the whole body or to specific organs.

In our study, the chromosome damage assessed showed a high dose dependent increase of the number of DC and MN during the therapy course. This increase appears more evident in patients completing all ^223^Ra six cycles.

In addition, a large DCs increase was unexpectedly observed in two patients between T7 and T30, not due to a supplementary ^223^Ra dose. This result suggests that PBLs could be exposed to an extra dose by the radiation emission from the target organs, highlighting possible adverse effects to non-target organs related to this type of therapy.

This hypothesis seems to be supported by the progressive increase in the number of cells with two or more DC, observed in all patients. These data indicate that chromosome damage accumulates in PBLs over time and reaches the highest complexity after the end of the therapy (T180), suggesting a persistence of the emission of alpha particles from the target to non-target organs.

It is noteworthy that the average background frequencies of chromosome damage (especially for dicentrics) found in PBLs of patients in T0 are very high if compared to background values found in healthy control subjects. This is more evident in patients previously treated with radiotherapy suggesting that patients treated with α-emitters, who underwent radiotherapies, should require particular attention on side effects to healthy tissues. In these patients, the radiation induced biological effect could, in fact, persist and could accumulate in PBLs for several years. T-lymphocytes are long-lived circulating cells that can be considered as circulating dosimeters and, among them, the population of long-lived lymphocytes has half-life of 3.5 years or more [24].

The number of DC and MN observed in the PBLs has been plotted against the AD to blood. A linear correlation (increase) has been found between AD to blood and DC number as reported for other radionuclides [11, 18, 31].

A proper biological dosimetrical approach, that is currently lacking, could improve treatment of patients with α-emitters in relevant aspects of radiation protection for decreasing the stochastic radiation induced effects or reducing the rate of secondary tumors.

However, the correlation between the delivered dose to blood and the information provided by biological assays strictly depends on selection of the appropriate calibration curves, that are usually generated in vitro using only external photon radiation. Thus, an in vitro dose-response curve for DC and MN induced by ^223^Ra should be developed, to compare the DC and MN frequency to the AD to blood at the corresponding times during therapy [32].

The average background frequency of MN in T0 is also very high (0.064 ± 0.016) compared with the background frequency in healthy population (quite variable from 0 to 0.040, depending on factors such as dietary, age and gender). On the other hand, the data is in line with the work of Lee et al. [33], in which the background frequency of MN in PBLs from prostate cancer patients undergoing radiotherapy is 0.057 ± 0.008. The background frequency observed in this study could therefore be due to previous therapy treatments, as observed for dicentrics.

Probably, this decrease could indicate a partial recovery of the damage as observed by MN, not maintained however in subsequent treatments.

Finally, ^223^Ra 3D-images could potentially improve the estimation of AD distribution and local RBE.

## Conclusions

The results of this study, despite the small sample of patients, highlight some interesting ideas that need to be further investigated: dosimetry may be useful to identify a more appropriate ^223^Ra administered activity predicting AD to target tissue; a dose dependent complex chromosome damage occurs during ^223^Ra administration that is more evident in heavily pre-treated patients; AD to blood could be used for radioprotection purposes.

## Supplementary Information

**Additional file 1: Fig. S1**.

**Additional file 2: Supplementary notes**.

## Data Availability

The datasets used and/or analyzed during the current study are available from the corresponding author on reasonable request.

## Abbreviations

AD: Adsorbed dose
ALP: Alkaline phosphatase
BS: Bone scan
CR: Complete
DC: Dicentric
EBRT: External beam radiotherapy
HB: Hemoglobin
LET: Linear energy transfer
mCRPC: Metastatic castration resistant prostate cancer patient
MN: Micronuclei
NCI-CTCAE: National Cancer Institute-Common Terminology Criteria for Adverse Events
OCR: Overall clinical response
PBLs: Peripheral blood lymphocytes
PSA: Prostate-specific antigen
PLT: Platelet
PR: Partial
RBC: Red Blood Cell Count
SAEs: Serious Adverse Events
SD: Stable
PD: Progressive disease
TTR: Target tissue response
T0: Baseline
T180: At 180 days
T30: At 30 days
T7: At 7 days
WBC: White blood cell count
