# Supplementary material for: The role of dosimetry and biological effects in metastatic castration–resistant prostate cancer (mCRPC) patients treated with 223Ra: first in human study
Source: J Exp Clin Cancer Res. 2021 Sep 6;40:281. doi: 10.1186/s13046-021-02056-9 (PMC8420003; doi:10.1186/s13046-021-02056-9)

**Supplementary:**

**Figure 1S**: Illustration of SPECT calibration procedure evaluating the scanner sensitivity, transmission and recovery coefficients.


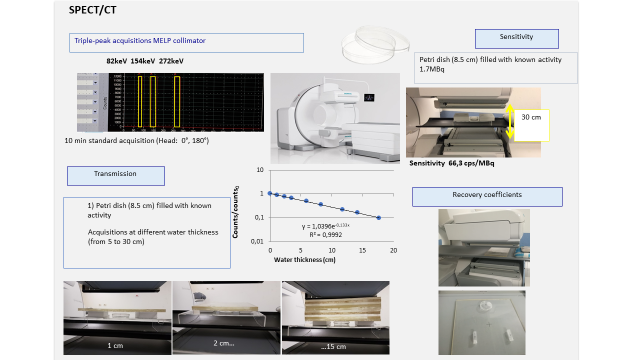

Supplement: Supplementary file 1 — Additional file 1: Fig. S1. [file 13046_2021_2056_MOESM1_ESM.docx]
