# Supplementary material for: The role of dosimetry and biological effects in metastatic castration–resistant prostate cancer (mCRPC) patients treated with 223Ra: first in human study
Source: J Exp Clin Cancer Res. 2021 Sep 6;40:281. doi: 10.1186/s13046-021-02056-9 (PMC8420003; doi:10.1186/s13046-021-02056-9)
Supplement: Supplementary file 2 — Additional file 2: Supplementary notes. [file 13046_2021_2056_MOESM2_ESM.docx]

**Supplementary notes:**

The energy window setting was chosen according to procedure of Hindorf et al. [^[[1]](#endnote-1)^] and all acquisitions were performed using a triple-peak acquisitions at 82, 154 and 272 keV with an energy window of 20%. SPECT calibration was performed evaluating the scanner sensitivity, transmission and recovery coefficients as summarized in **Fig.1S** (see supplementary data).

Sensitivity was carried out using a Petri dish (8.5 cm) filled with known activity 1.7 MBq placed on the couch in the center of the field of view (FOV) at 15 cm of distance from each head as suggested in [^1^]. Sensitivity was measured using 5 vials with different areas for all the acquired energy peaks (i.e. 82, 154 and 272 keV).

Attenuation correction factor was determined using a point-like source placed in the center of the FOV filled with known activity placed on the couch placing different solid water phantom thicknesses (from 1 to 30 cm). The effective mass attenuation coefficient $\text{μ/ρ}$ was obtained as the slope of the measured curve (i.e. counts at different thickness divided by the extrapolated counts at zero thickness versus thickness d), using the following equation:

$$\frac{\text{μ}}{\text{ρ}}\text{=-}\frac{\text{d}}{\text{ρ}}\text{*}ln\frac{I\left( x \right)}{\text{I}\left( 0 \right)}$$

where $\text{I(x)}$ is the acquired number of counts for the energy window setting E when d cm solid water was inserted between the source and collimator, $\text{I(0)}$is the number of counts in air for the energy window setting and $\text{ρ}$ is the density.

The effective mass attenuation coefficient for water was assumed equal to the effective mass attenuation coefficient for solid water phantom having a density of 1.062 g/cm^3^. The effective mass attenuation coefficient was determined for each energy peak using the solid water phantom and its value was compared with the theoretical value ([https://www.nist.gov/](about:blank))

The spatial resolution was determined as the FWHM of a gaussian interpolation of the point-spread function according to Hindorff et al. [^1^].

To assess the partial volume effect, a known amount of ^223^Ra was uniformly distributed in several rectangular or circular vials and imaged in air with a source-to-collimator distance of 15 cm. All the vials contained the same activity concentration, respectively, which were filled with the same liquid thickness according to the different basal area (the phantom with the largest radius contained the highest activity). Regions of interest (ROIs) of the known phantom diameters were applied to the images to evaluate the spill-out of counts from these regions.

Recovery coefficients were calculated determining the ratio between the measured counts and the theoretical activity (i.e. expected activity concentration measured during the vial preparation). The recovery coefficients were determined for various vial areas and energy peaks.

The distance between the patient and the collimators was chosen at the first acquisition and was kept fixed during the biokinetics study. The equivalent thickness for evaluating the local attenuation was calculated based on CT study of PET/CT acquisition according to Siddon procedure [^[[2]](#endnote-2)^]. After each measurement session, the response stability of the gamma cameras was checked with static acquisitions of a test activity.

1. Hindorf C, Chittenden SJ, Aksnes A-K, Parker C, Flux GD. Quantitativeimaging of 223Ra-chloride (Alpharadin) for targeted alpha-emittingradionuclide therapy of bone metastases. Nucl Med Commun. 2012;33:726–32. [↑](#endnote-ref-1)
2. Siddon RL. Fast calculation of the exact radiological path for a three-dimensional CT array. Med Phys. 1985;12(2):252–255 [↑](#endnote-ref-2)
